# Double-Ended Calibration of Fiber-Optic Raman Spectra Distributed Temperature Sensing Data

**DOI:** 10.3390/s120505471

**Published:** 2012-04-27

**Authors:** Nick van de Giesen, Susan C. Steele-Dunne, Jop Jansen, Olivier Hoes, Mark B. Hausner, Scott Tyler, John Selker

**Affiliations:** 1 Department of Water Management, Delft University of Technology, Stevinweg 1, CN Delft 2628, The Netherlands; E-Mails: S.C.Steele-Dunne@tudelft.nl (S.C.S.-D.); jhamjansen@gmail.com (J.J.); o.a.c.hoes@tudelft.nl (O.H.); 2 Department of Geologic Sciences and Engineering, University of Nevada, Reno, MS 172, Reno, NV 89557, USA; E-Mails: mhausner@unr.edu (M.B.H.); styler@unr.edu (S.T.); 3 Department of Biological and Ecological Engineering, Oregon State University, 116 Gilmore Hall, Corvallis, OR 97331, USA; E-Mail: john.selker@oregonstate.edu

**Keywords:** Distributed Temperature Sensing (DTS), calibration, environmental monitoring, hydrology

## Abstract

Over the past five years, Distributed Temperature Sensing (DTS) along fiber optic cables using Raman backscattering has become an important tool in the environmental sciences. Many environmental applications of DTS demand very accurate temperature measurements, with typical RMSE < 0.1 K. The aim of this paper is to describe and clarify the advantages and disadvantages of double-ended calibration to achieve such accuracy under field conditions. By measuring backscatter from both ends of the fiber optic cable, one can redress the effects of differential attenuation, as caused by bends, splices, and connectors. The methodological principles behind the double-ended calibration are presented, together with a set of practical considerations for field deployment. The results from a field experiment are presented, which show that with double-ended calibration good accuracies can be attained in the field.

## 1. Introduction

In recent years, Distributed Temperature Sensing (DTS) based on Raman backscatter from fiber optic cables has found extensive environmental applications [1,2]. A DTS instrument sends a short laser pulse into an optical fiber and measures photons scattered back from within the fiber. From the time of flight, the position of the scattering element in the fiber can be calculated. Most backscattered photons have the same frequency as the original laser pulse. Some, however, will show the effect of Raman scattering, with some having a lower frequency (called Stokes) and others a higher frequency (called anti-Stokes). The intensity of the anti-Stokes backscatter is very sensitive to the temperature of the scattering element while the Stokes backscatter is much less sensitive. The ratio of the two then allows for the calculation of the temperature of the fiber. The advent of new DTS applications was made possible by the development of robust fiber optic cables and improved specifications and lower costs of DTS systems. With good calibration, even less expensive DTS systems provide 1–2 m resolutions along cables of up to 5 km, with 0.1 K accuracies for integration times of 60 s. Applications started with dam surveillance [3] and borehole observations [4] and have multiplied rapidly to include monitoring of ice caves [5], estuaries [6], sewers [7], solar ponds [8], soils [9–11], groundwater [12,13], streams [14–17], lakes [18], atmosphere [19,20], electric transmission cables [21], mines [22], and gas pipelines [23].

DTS systems tend to be relatively user friendly plug-and-play systems that immediately provide temperature profiles. What is not always clear is how the temperature profiles are derived from the Stokes and anti-Stokes components of the Raman backscatter by the DTS system, and what defects in these measurements might be present. Often internal reference temperatures are used to determine a temperature offset. Stokes and anti-Stokes have different extinction coefficients and assumptions have to be made about this differential attenuation along the cable. Sometimes spatial filtering techniques are used that hide sudden temperature changes along the cable. Connector, cable, and splices all have a priori unknown effects on Stokes and anti-Stokes backscatter. Depending on the exact application, users may want to have more control over the determination of the temperature and to calibrate temperature profiles themselves to achieve the highest possible accuracy.

In the companion paper [24], calibration techniques that can be applied for “single-ended” measurements are presented. In this context, single-ended measurements refer to those measurements in which laser pulses are both injected and monitored at only one end of the fiber optic cable. For double-ended measurements, on which we focus here, pulses are injected and monitored from both sides, alternating after each measurement interval. The main motivation for double ended measurements is that it allows for direct calculation of the differential attenuation along the cable. Differential attenuation refers to different absorption rates of Stokes and anti-Stokes along the optical path, which may erroneously be interpreted as temperature signals. For homogenous cables that are not spliced, stressed, or bent, this may not be required, but when but when such conditions exist, direct observation of the differential attenuation is almost essential to obtaining accurate measurements. Beyond facilitating calibration under typical field conditions, unlike single ended methods, double ended set-ups allow all calibration baths to be located adjacent to the DTS apparatus, where power and shelter are already present. For some applications, such as down oil or water wells, it is not practical to place a bath at the end of the cable, making a double ended measurement necessary [25]. The main disadvantages are a somewhat more complicated installation, added algorithmic complexity, and often a noisier signal near the DTS apparatus. The central issue addressed in this paper is the extent to which double ended measurements can be used to correct for differential attenuation in environmental applications. As such, the paper is also of relevance for a new type of DTS that has recently been put forward, based on double-ended anti-Stokes measurements only (backscatter and forward scatter) [26].

Hausner *et al.* [24] contains an in-depth description of the functioning and physics of the type of Raman spectroscopy used by most DTS systems. In this paper, we build on those descriptions and add, in addition to the double-ended calibration approach, some details concerning construction and operation of calibration baths. The primary advantage for double-ended calibration method in environmental applications is that this method only requires calibration baths near the DTS instrument, which is useful in cases where it is not possible or practical to maintain calibration baths along the fiber optic cable. However, no detailed description of double-ended DTS calibration for environmental applications is available in the literature, and this technique is now widely used by practitioners. This article fills this gap and provides researchers with a calibration protocol, a set of practical considerations for the application of this method in the field, and a case study that highlights the advantages and disadvantages of this method.

## 2. Experimental Section

### 2.1. Determining Differential Attenuation

From basic principles, the temperature of an optical fiber may be determined by the ratio of the Stokes and anti-Stokes backscattered light using the following equation [24,27]:

(1) $$T(z,t)=\frac{\gamma }{\text{ln}\left(\frac{P_{S}(z,t)}{P_{aS}(z,t)}\right)+C(t)-\int_{0}^{z}\Delta \alpha (z^{\prime })dz^{\prime }}$$

where *T* is the temperature (K) and *z* is the distance along the cable with *z* = 0 at the DTS instrument. The ratio *P_S_*(*z,t*)/*P_aS_*(*z,t*) is the measured ratio between the power of the Stokes (*S*) and anti-Stokes (*aS*) backscatter reaching the instrument. The integral in the denominator represents the product of length and the differential attenuation of the Stokes and anti-Stokes radiation caused by the differences in the absorption coefficients for both frequencies. In this manuscript, we term this dimensionless integral the “cumulative differential attenuation”. In practice, the remaining terms need to be calibrated. Theoretically, the numerator *γ* (K) depends on the distribution of quantum states, *γ*=*ħ* Ω/*k*, with *ħ* being Planck's constant, *Ω* the difference in frequency between the backscattered Stokes radiation and the incoming laser pulse, and *k* Boltzmann's constant. A typical 1064 nm laser, with *Ω* ≈ 10^13^ Hz, gives *γ* ≈ 490 K [27]. In practice, *γ* is often treated as a constant for a given DTS system although the complete system of laser, gratings or prisms, and photon detectors will cause minor changes in *Ω* as function of instrument temperature and power supply [24]. In our case, we work with the value *γ* = 482.1 K, the optimal value over the measurement period. The term *C(t)* accounts for the differences in effective detector sensitivities with respect to Stokes and anti-Stokes photons, which may vary in time. Temporal changes in *C*(*t*) may be caused by thermal sensitivity of the detectors, as well as thermal variation in the alignment of the optical system, which all can result in changes in *C*(*t*). This makes *C*(*t*) dependent on, among other variables, (changes in) instrument temperature. Since *C*(*t*) is not a function of position along the cable, its calculation does not require the special data from a double-ended installation.

The term *Δα*(*z*) represents the differential attenuation of the Stokes and anti-Stokes backscatter along the cable. The extinction coefficients of Stokes and anti-Stokes radiation are not equal, which has the effect that the ratio *P_S_*(*z,t*)/*P_aS_*(*z,t*) observed at the DTS unit is not equal to the ratio at the point along the cable where one wants to measure the temperature. The net effect that differential attenuation has on the value of the denominator of Equation 1 needs to be compensated in order to obtain the correct temperatures. The effect is cumulative, that is to say all the differential attenuation effects between the DTS unit (*z′* = 0) and the point of interest (*z′* = *z*) need to be integrated. The differential attenuation is often expressed in decibel (dB, 10 times logarithm to base 10) per meter or per kilometer. The integral $\int_{0}^{z}\Delta \alpha (z^{\prime })dz^{\prime }$ then has the unit of dB but this needs to be scaled with 0.1 × ln(10) to compensate the denominator term ln(*P_S_*(*z,t*)/*P_aS_*(*z,t*))directly. When the differential attenuation is uniform along a cable, the value of *Δα*(*z*) in the denominator is constant and the integral in the denominator simplifies to *Δα*·*z*. In such a case, *Δα* can be determined through calibration based on additional independent temperature measurements at two points along the cable. In environmental sensing applications, however, there are often changes in *Δα* along the cable, associated with bends, connectors, splices and other irregularities. Note that attenuation in connectors can be a function of temperature, pointing out the possible temporal variation in this parameter. In general, one does not expect *Δα* to change rapidly in time if the cable's position and environment does not change rapidly. In the case of spatially non-uniform *Δα*, the integral can be computed based on double ended measurements, which is the main topic of this paper.

For a double ended measurement, pulses are launched into the fiber from one end of the cable during a first measurement period, after which pulses are launched into the other end during the next measurement period. We will call the two directions ‘forward’, starting at *z* = 0, and “reverse”, starting at *z* = *L*, with *L* being the length of the cable. If we can assume that the temperature of a small stretch, *Δz*, of the optical fiber remains constant in time during the forward and reverse measurement, we can use Equation 1 to solve for the differential attenuation, *Δα*, over *Δz*:

(2) $$T(z)=\frac{\gamma }{\text{ln}{\left(\frac{P_{S}(z)}{P_{aS}(z)}\right)}_{\Rightarrow }+C-\int_{0}^{z}\Delta \alpha (z^{\prime })dz^{\prime }}$$

(3) $$T(L-z)=\frac{\gamma }{\text{ln}{\left(\frac{P_{S}(z)}{P_{aS}(z)}\right)}_{⇐}+C-\int_{L}^{L-z}\Delta \alpha (z^{\prime })dz^{\prime }}$$

The arrows in the equations indicate whether the measurement is forward (⇒) or reverse (⇐). By setting *T*(*z*) = *T*(*L*−*z*) and after some algebra, we obtain:

(4) $$\int_{z}^{z+\Delta z}\Delta \alpha (z^{\prime })dz^{\prime }=\frac{\text{ln}{\left(\frac{P_{S}(z+\Delta z)}{P_{aS}(z+\Delta z)}\right)}_{\Rightarrow }-\text{ln}{\left(\frac{P_{S}(z)}{P_{aS}(z)}\right)}_{\Rightarrow }+\text{ln}{\left(\frac{P_{S}(z)}{P_{aS}(z)}\right)}_{⇐}-\text{ln}{\left(\frac{P_{S}(z+\Delta z)}{P_{aS}(z+\Delta z)}\right)}_{⇐}}{2}$$

By stepping along the complete cable with steps of *Δz*, which are normally set equal to the spatial measurement interval, one obtains the value of $\int_{z}^{z+\Delta z}\Delta \alpha (z^{\prime })dz^{\prime }$ for each interval *Δz*. If we sum these values, starting at *z* = 0, we obtain the estimate of $\int_{0}^{z}\Delta \alpha (z^{\prime })dz^{\prime }$ for every *z*. A key observation here is that the data used to obtain these values can be obtained from a time-average over the entire period during which the differential attenuation was considered to be constant. Employing a much longer period of integration than is to be used in a typical measurement reduces the noise of the values employed in the calibration, thus typically allowing these calibration values to be sufficiently low in pure noise as to not to appreciably add to the intrinsic uncertainty of the estimated temperature that is due to statistical variability in photon count. If the differential attenuation is further seen to vary smoothly in space, which is typically the case outside of defects and splices, then noise can be reduced through spatial averaging.

### 2.2. Experimental Set-Up

Measurements were taken from 10 through 12 November 2009, in and near the large (2 ha) stilling pond of a pumping station in Delft, The Netherlands (51°59′5″N, 4°22′43″E). The DTS instrument was a HALO unit (Sensornet, Elstree, UK). The HALO unit was selected solely for the reason that this instrument was the only available instrument at the time within the Delft research group. The HALO is a relatively inexpensive instrument. This instrument has specifications in terms of signal-to noise ratio and/or power consumption that are not as good as higher end DTS instruments but which are sufficient for many applications, including the present demonstration of double-ended calibration. For a more in-depth overview of important performance indicators of DTS instruments, we refer to [2]. The spatial sampling resolution of the HALO is 2 m, suggesting a >4 m spatial resolution based on the Nyquist criterion, and the factory specified resolution was 0.07 K at a 1 km cable length for measurement times of 60 s. The cable used consisted of two (duplex) graded-index multi-mode optical fibers, loosely enclosed in a gel-filled polybutylene terephthalate tube (PBT). The PBT tube was protected by a stainless steel coil, a layer of Kevlar™ strands, and a plastic jacket (Kaiphone, New Taipan City, Taiwan). The one-way length of the cable was about 770 m. The unit was programmed in such a way that measurements were taken for 30.05 s. Then the unit switched to the alternate channel and measured another 30.05 s from the other end of the fiber. Because the two fibers were spliced at the end of the cable to form one long fiber, it was possible to make double ended measurements. Such a splice is not necessary; one could also simply loop one cable back to the unit. Though this setup included two measurements at each point due to the duplex cable construction, a double ended measurement in general does not need to be duplexed. Refer to Hausner *et al.* [24] for making use of duplexing in DTS calibration.

**Figure 1.**
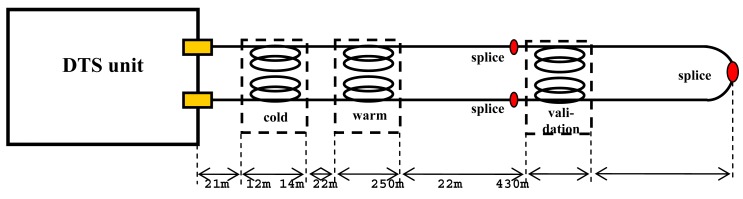
Schematic set-up for calibration test. Lengths are not to scale. The forward and reverse fibers were enclosed in a PBT tube (duplex cable). Between warm bath and first splice and between validation bath and end splice, the cable was lying at the bottom of a water body.

Three baths were used: a cold and a warm bath near the DTS unit and a validation bath near the middle of the cable. All baths and the DTS unit were placed inside the pumping station building with a relatively stable temperature of around 10 °C ± 2 °C. The cable was led from the pumping station into the stilling pond, where it rested at the sediment-water interface (Figure 1). Inside the pond, the cable was not mechanically fixed to the bottom but rested under its own weight because flow velocities were extremely low (<0.01 m/s). Between the DTS instrument and the pond, the cable was fixed with duct tape while making sure that no impingements or sharp bends occurred. The cable lengths were: 21 m DTS to cold bath, 12 m inside cold bath, 14 m cold to warm bath, 22 m inside warm bath, 250 m warm bath to validation bath, 22 m inside validation bath, and 430 m from validation bath to end splice. A total of three splices were made; one at the end of the cable connecting the two individual optical fibers to allow for double ended measurements; and one in each fiber just before the validation bath to connect two cable pieces. Each splice was fixed by carefully taping the splice lengthwise to a rigid plank. Because this trial only lasted a few days, no special housing for the splices was used, which would be needed for longer experiments (>1 week). The splice at the end of the cable was placed outside the water on land. The other two splices were indoors with the DTS unit and the baths.

The standard way to ensure that the cable has a known temperature over a given length is to coil the cable and put the coil into a water or ice bath inside a large insulated container, such as a cooler used to protect foods. For this experiment, we used a cooler with internal dimensions of 0.4 m high, 0.55 m long and 0.4 m high. Care should be taken that the cable does not rest on the bottom or touch the sides of such a container, where the temperatures are likely to be distorted by wall conduction. Ideally, the bath temperatures would bracket the full range of temperatures that can be expected to occur along the cable, though since the system is linear in its calibration coefficients this is not strictly required. If ample electrical energy is available, a warm bath is not difficult to create using an aquarium heater. Keeping a bath below ambient temperature in the field is typically more challenging. Melting ice made from pure water in a bath of pure water establishes a temperature of 273.25 K under normal atmospheric pressure. Water with ice floating in it does not generally have a 273.25 K temperature since the greatest density of water occurs around 277 K. Therefore, the bath will typically stratify with near 273 K at the surface, and near 277 K at the bottom of the bath. Thus, it is essential that such a bath have water-wetted ice extending below the depth of the cable under test. With these aspects in mind, it is not surprising that it is difficult to maintain a useful ice bath in the field. An alternative approach to maintaining a certain temperature is to accurately monitor the temperature of a well-insulated, well mixed bath so that the reference bath temperature is well-known at any time [e.g., as needed for the computation of *C*(*t*)], though possibly slowly changing in time. For full calibration, two distinct temperatures are required. For long term deployment, where continuous maintenance of an ice bath is not possible, one can employ a single Peltier junction heat-pump situated between two baths to ensure that there will always be one cold and one warm bath. Such a system was employed in the experiments described here. The complexity added to the set-up through the addition of the Peltier junction will only be worthwhile in special cases. When energy supply is a limiting factor, one can use passive heating and cooling by covering the coolers with insulating materials that have different thermal and/or radiative properties. In all cases, it is important to avoid stratification, with an easy solution being the installation of a continuously running aquarium bubbler outlet at the bottom of the bath.

The temperature of the baths should be continuously recorded. Many sensors exist that can monitor temperature to within 0.1 K, and some field-portable units are accurate to 0.002 K (e.g., the Fluke 1524). The standard sensor would be a PT100 (100 Ohm platinum resistance thermistor) with a logger capable of a four point resistance measurement. Here, we used a cheaper alternative, the TSic 506 (ZMD, Dresden, Germany), which produces a digital output and has a factory guaranteed accuracy of 0.2 K. Here, we used a set of four TSic 506 per bath. An offset correction for each TSic 506 was determined by monitoring them in a triple-point cell for water (273.16 K). The resulting accuracy of the bath temperature was within 0.04 K.

### 2.3. Calibration

DTS units typically provide “raw” output of temperature traces that are referenced to a single internal calibration coil. These values are based on default assumptions concerning connector losses, differential attenuation in the external fiber, and equilibrium between the internal reference coil and the sensor monitoring it, which typically introduce time-varying errors that exceed 1 K, with spatial and temporal variation, and thus are inadequate for most environmental sensing applications.

Continuous, installation-specific calibration is recommended, which can be understood by considering Equation 1 illustrating that Stokes and anti-Stokes backscatter may vary over time and space. The calibration process chosen here takes *γ* as a constant, *C*(*t*) as a function of time only, and *Δα*(*z*) as a function of position only. The assumptions concerning *γ* and *C*(*t*) are physically plausible, so in the calibration protocol we apply them directly. It may happen, however, that *γ* does show variation over time and that better results are obtained when both *γ* and *C*(*t*) are calibrated for [24]. We also assume that *Δα* does not vary over time. Again, in practice, this is not always true. Especially when straining and bending varies over time, which is often the case in atmospheric DTS applications, or when mechanical fiber junctions are exposed to varying temperatures, this assumption can lead to poor performance. It should be noted that although for this experiment we assume *Δα* only varies over space, that if there is reason to believe there may be slow changes over time in *Δα*, one may employ the techniques provided here but recalculating *Δα* over time intervals over which the values may be taken as constant. One may even opt for the entirely feasible option of re-computing *Δα* for each measurement, although this tends to increase the noise in the resulting temperature data [24].

The calibration algorithm first calculates *Δα*(*z*), using Equation 4 for each time step, after which *Δα*(*z*) is averaged over time. The calculated *Δα*(*z*) for single time steps varied over space from one point to the next, also over homogenous stretches of optical fiber. The main reason behind this result is that subtraction tends to increase noise levels. Because there is no physical ground to assume such random variability over space from one point to the next, a smoothing in the form of a simple piece-wise linear regression was applied over stretches between splices that could be assumed to be homogenous.

Once the integrated *Δα*(*z*) is computed, the remaining unknowns, *γ* and *C*(*t*), can be estimated. For each time step, Equation 1 is used to minimize the square error between calculated DTS temperatures and independently measured temperatures in the cold and warm baths. In the analysis provided here, during a first approach, both *γ* and *C*(*t*) were allowed to vary. This resulted in highly correlated values of *γ* and *C*(*t*). In a second approach, *γ* was fixed at the average value of the first run (482.1 K), after which only *C*(*t*) was allowed to vary. The difference in Root Mean Square Error (RMSE) obtained between time varying and fixed *γ* was very small (0.005 K), so for this application a fixed value of *γ* provided acceptable accuracy and reduced the number of calibrated parameters.

## 3. Results and Discussion

### 3.1. Differential Attenuation

**Figure 2.**
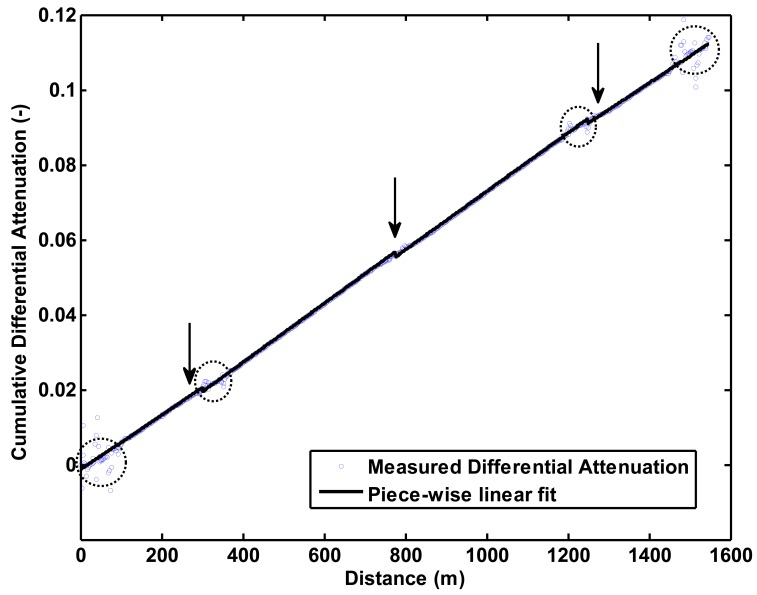
Measured Cumulative Differential Attenuation as calculated with Equation (4) and piece-wise linear fit. The arrows indicate the location of the splices and the dotted ellipses indicate the location of the different baths.

Direct application of Equation 4 gives a fairly noisy result with respect to the differential attenuation along the cable, especially where sharp transitions in temperature and/or material (splices, bends) are found (Figure 2). The noisy result is caused by the differences employed in Equation 4, which amplify any noise present in the forward and reverse measurement. In order to obtain smoother results, a piece-wise linear fit was applied with breaks at those points where one would expect larger changes in differential attenuation. In this case, we placed the breaks at locations of the three splices. The noisy patches at the edges of the transitions where not included in the regression. The cables on both sides of the splice came from different production batches, which helps explain the different slopes in Figure 2. Also, the two fibers within one cable can have different slopes, as is the case in this experiment, especially in the cable stretch nearest the DTS unit. It is worth noting that this approach is functionally similar to the method in the companion paper [24] of correcting for each step loss and then assuming uniform *Δα*(*z*). The main difference is that in the case of double ended measurements, one does not need to measure temperatures independently at the site of the splices. In the case of boreholes and other places that are difficult access, this may be an advantage.

**Figure 3.**
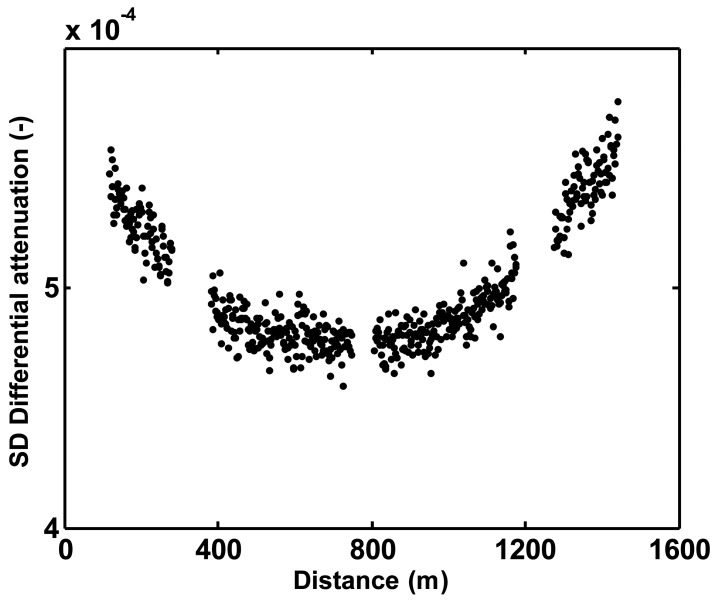
Standard deviation over time of differential attenuation calculated at each sampling interval along the cable as measured over the complete measurement period. Blank stretches coincide with sharp transitions and splices where the standard deviation was much larger than the values shown here.

Figure 3 shows an important disadvantage of double ended measurements. The further one moves away from the DTS unit, the fewer backscattered photons are returned to the sensing unit (Beer's Law), reducing the signal to noise ratio. The noise increases exponentially with distance. A point that is close to the DTS in one measurement direction is far from the DTS when the measurement is taken from the other side of the fiber. When these measurements are used to calculate differential attenuation, using Equation 4, the noisy *P_S_*(*z,t*)/*P_aS_*(*z,t*) measurements translate into noise in the calculated differential attenuation. The lowest noise level in differential attenuation is, therefore at the middle of the cable. Especially for applications with longer cables, this becomes very relevant. If at all possible, long integration times should be employed in developing the data used to calculate the differential attenuation so that the noise in this term introduces less uncertainty than the intrinsic noise of an individual measurement. The use of smooth functions to describe the differential attenuation rather than the actual point-to-point computed value also can reduce this noise, and is generally reasonable in a continuous section of fiber where attenuation should vary little in space.

The default differential attenuation term from the DTS manufacturer was 0.42 dB/km, whereas the average measured values were 0.64 dB/km. Such an error will have a very large effect on the temperature estimates without double ended measurements or calibration baths. A quick check of Equation (1) shows that for temperatures near 300 K, such as in this experiment, the denominator must be about 1.6. After one kilometer, the difference between default and actual differential attenuation would be 0.22 dB, which corresponds with a change in the denominator of about 0.05, or an error of 3% in absolute temperature, or 10 K.

### 3.2. Accuracy Assessment

**Figure 4.**
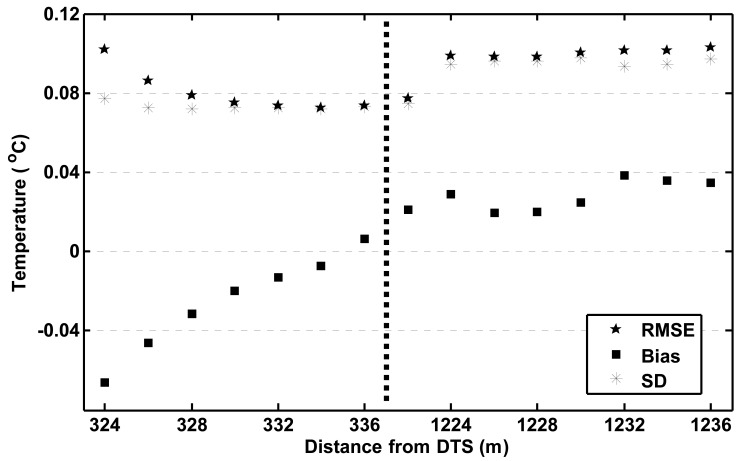
Root Mean Square Error (RMSE), Bias, and Standard Deviation (SD) for measurements in validation bath. The dotted line separates points before and after the splice at the end of the cable.

The measurement accuracy was assessed by comparing the DTS measurements in the validation bath with the TSic 506 measurements in the same bath. In total, there were 15 DTS measurement points in the validation bath. For each point, the RMSE and the bias were calculated, whereby the TSic 506 measurements were taken as the “true” temperatures. Figure 4 shows the results. The average RMSE of the points before the splice, from 326 m to 338 m, was 0.080 K. The RMSE of the points after the splice, from 1,224 m to 1,236 m, was 0.10 K. The overall RMSE of the bath temperature, based on the average of all 15 points, was 0.060 K and the bias was 0.003 K. Under laboratory circumstances, Voigt *et al.* [28] found a standard deviation that was very close to the factory supplied specification of 0.07 K for measurement integration times of 60 s at 1 km. Here, we find slightly higher values than specified by the factory but this may be partially due to the fact that the accuracy of the TSic 506 measurement was around 0.04 K. We may conclude that also under these field conditions, one can obtain temperature measurements with accuracy close to the nominal one after careful calibration. The RMSE for the cold calibration bath was 0.047 K, and for the warm bath 0.029 K. Although the present measurements are not sufficient to obtain a detailed distance-accuracy relation, the increase in RMSE of 0.02 K between 330 m and 1,230 m is consistent with the expected exponential increase of RMSE with distance as specified by the manufacturer. Assuming such exponential relation is indeed valid, the RMSE for the present set-up would increase to 0.15 K at 2,000 m, and 0.32 K at 4,000 m.

Further analysis shows two additional interesting facts. First, spatial averaging over the 15 points in the validation bath does not reduce the variance to the extent as it would if the errors between adjacent points were completely independent. The variance (MSE-Bias^2^) for normally i.i.d. errors would scale with the number of observations. The associated Standard Deviation (√variance) would scale with the square root of the number of observations. In this case, however, the reduction is equivalent to the reduction in variance one would expect with about four independent measurements. The sample spacing appears to be about half the actual spatial resolution, consistent with optimal Nyquist sampling. This is quite typical of DTS systems, so users should be careful to distinguish between spatial sampling and spatial resolution.

The second point of interest is that the bias is very sensitive to the exact values of the differential attenuation correction, but the variance is not. Note that Figure 4 suggests that there is a structure to the bias in the validation bath before the splice, as it goes up from −0.07 °C to +0.02 °C. It is not clear what causes this structure or if it is simply a coincidence. A simple sensitivity analysis showed that this structure is not affected or caused by the differential attenuation correction so this is not likely to be the underlying cause. The bias values, however, are strongly affected, even by very minor changes in the differential attenuation correction. This underlines the importance of a robust estimation of the correction.

### 3.3. Effects of Shifts and Chromatic Dispersion

**Figure 5.**
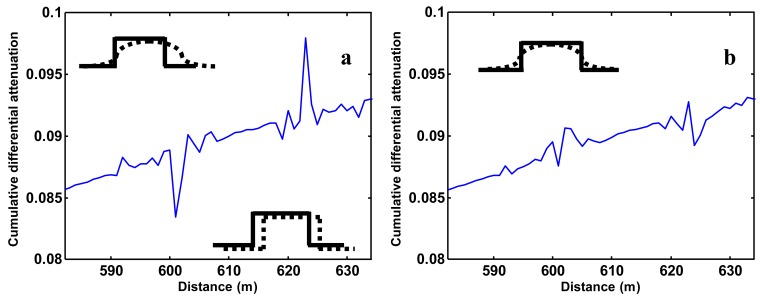
The schematic drawings show the nearby forward anti-Stokes (continuous line) and the shifted and more dispersed far reverse anti-Stokes (dotted line) responses. (**a**) Overshoot due to misalignment; (**b**) Overshoot due to dispersion.

One effect that has not been addressed yet, but became clear during the analysis, is the effect of shifts and chromatic dispersion. This effect should not be confused with path-length achromatic dispersion, caused by different path lengths for different modes in a multi-mode fiber and which is not present in single-mode fibers. Chromatic dispersion occurs equally in single-mode and multi-mode systems. The speed of light in a medium, such as an optical fiber, is wavelength dependent. Being another hidden calibration parameter, each DTS unit must determine the difference between the light velocities (chromatic dispersion) of the Stokes and anti-Stokes light which is a function of the index of refraction of the glass employed in the cable. The algorithms employed by different DTS manufacturers are quite different, with some machines being prone to errors in the chromatic dispersion corrections. Even a small error may, after a substantial length, cause substantial problems since the calculation of backscatter ratio will effectively be combining Stokes and anti-Stokes data from different locations along the fiber. These errors are most evident near abrupt changes in temperature, where an “S” shape error will result at the beginning and end of the temperature change (Figure 5(a)). It should be further realized that the measurements represent overlapping sampling intervals (recall the true spatial resolution is on the order of 4 m). In addition, it is likely that we have overlapping sampling intervals when starting at the different ends of the cable, unless the fiber is an exact multiple of the spatial sampling interval. With this in mind, we resampled the measurements and shifted the anti-Stokes measurement through a simple weighted linear interpolation to 0.55 of the next measurement interval in the baths. The result is shown in Figure 5(b) where the overshoot is largely removed.

After the correction, some “ringing” remains where sharp temperature transitions occur, but with intervals of less than the 4 m resolution of the instrument. Multi-mode fiber allows for light paths of slightly different length, which limits the bandwidth of data transfer compared to single mode fiber, but this is on the order of 10 cm dispersion per km, so does not explain the effects shown in Figure 5. Such achromatic dispersion will smooth pulses, as indicated (with exaggeration) in Figure 5(b). The differential attenuation is calculated by comparing forward and reverse signals. In the case when the transition is nearby for the forward pulses, in our case at about 600 m, the photons backscattered from the forward pulse travel a shorter distance through the fiber than the photons backscattered from the reverse pulses. The forward backscatter signal will then show less achromatic dispersion than the reverse backscatter signal (Figure 5(b)).

As shown here, ringing may occur for a certain instrument and cable set-up around sharp temperature transitions. It is important to leave ample cable between a warm and cold bath to assure that the measurements do not influence each other, and to verify that the data near temperature transitions is not used in calibration. Thus, it is important to be conservative in estimation of the length of cable needed in the baths to ensure a desired number of valid measurements. The sampling interval of the HALO used in this experiment is 2 m, but distance between two independent measurements is much larger. Typically, ensuring at least ten independent points are in the bath is a safe rule-of-thumb estimate of the cable length needed in one bath. The notion here is that the uncertainty in the actual measurement from 10 independent points should be about √1/10 ≈ 1/3 of the uncertainty found in any point along the rest of the cable. Since the calibration procedure will incorporate this uncertainty in the final computed values, the idea is to have the calibration baths long enough so that the uncertainty in calibration parameters does not greatly increase the overall uncertainty in reported temperatures. Keep in mind that temperatures from each of the baths will suffer from this issue, so even with 10 sample points in each bath, the uncertainty in calibration parameters approximately doubles the uncertainty in the final estimated temperatures (assuming that the calibration parameters are re-estimated at he measurement interval).

## 4. Conclusions

The main conclusion is that double ended calibration is practical, and provides many benefits, under field conditions. The accuracy obtained in a typical installation, as expressed by the RMSE, was found to be close to the measurement resolution specified by the manufacturer. The results in terms of accuracy are comparable to those found in the companion paper on single ended calibration techniques. The measurement of the actual differential attenuation has been shown to be critical for accurate absolute temperature measurements.

Among the cautions of employing double ended measurements is that the calculated differential attenuation will amplify temperature errors arising from errors in chromatic dispersion corrections, which are evidenced by “ringing” around sharp transitions. For this and many other reasons, the length of cable needed in the calibration baths should be estimated with care, and should in general be >10 times the DTS spatial resolution.

The calibrated value of differential attenuation introduces noise, even along a spatially uniform cable. Time averaging is essential in reducing this contribution to uncertainty, but even after almost two days of measurements, this noise was not negligible. Points at both ends of the fiber are most affected. By designing the experimental set-up such that the most critical observations are made around the center of the cable, one is least affected by noise in differential attenuation. From a practical point of view, one can further reduce the noise by employing spatial smoothing along sections of cable that have consistent properties.
